# Investigating factors affecting the prevalence of stress, anxiety and depression among citizens of Karaj city: A population-based cross-sectional study

**DOI:** 10.1016/j.heliyon.2023.e16901

**Published:** 2023-06-02

**Authors:** Sara Armandpishe, Reza Pakzad, Mohammadamin Jandaghian-Bidgoli, Fatemeh Abdi, Maryam Sardashti, Kimia Soltaniha

**Affiliations:** a Student Research Committee, Nursing and Midwifery Faculty, Alborz University of Medical Sciences, Karaj, Iran; b Student Research Committee, Faculty of Health, Ilam University of Medical Sciences, Ilam, Iran; c Student Research Committee, Nursing and Midwifery Faculty, Shahid Beheshti University of Medical Sciences, Tehran, Iran; d Assistant Professor, Non-communicable Diseases Research Center, Alborz University of Medical Sciences, Karaj, Iran

**Keywords:** Stress, Anxiety, Depression, Prevalence

## Abstract

**Background & aim:**

Stress, anxiety, and depression are among the major public health problems worldwide. These problems, which may lead to more challenges, continue to grow. Therefore, the aim of this study was to investigate the main factors affecting the prevalence of stress, anxiety, and depression among citizens of Karaj.

**Materials & methods:**

A total of 920 people participated in the study. The Depression, Anxiety, and Stress Scale (DASS), consisting of 21 items, was used for the assessment of the studied problems. Single and multiple regressions were used for the data analysis. In addition, all statistical analysis was done by Stata version 12 at the 0.05significance level.

**Results:**

We found that the prevalence rate of depression, anxiety, and stress was 4.79%, 13.28%, and 15.13%, respectively. Education level was significantly associated with stress (b: 1.21; p < 0.001), anxiety (b: 2.03; p < 0.001), and depression (b: 0.48; p: 0.039). The association of the female gender with stress (b: 2.05; p < 0.001) and anxiety (b: 1.01; p: 0.002) was also confirmed. The association of being divorced or widowed with stress (b: 1.84; p: 0.005), anxiety (b: 1.21; p: 0.001), and depression (b: 1.85; p: 0.003) was approved. There was also a significant association between family size and stress (b: 0.39; p: 0.041). Gender and economic status were known as the most effective factors in the incidence of the studied problems.

**Conclusion:**

The prevalence rate of depression, stress, and anxiety in Karaj should be taken into consideration. Therefore, the preventive and therapeutic measures need to be considered for reducing the effects of the risk factors.

## 1 Introduction

### 1.1 Background

Living in the current era and being exposed to social and economic stressors have increased the prevalence of mental problems [1]. Mental health is one of the most important aspects of the general health, that can have a serious impact on the physical, social, and spiritual condition of every individual. Mental health is the key to a healthy life [2]. Also, the importance of mental health becomes doubly significant when it is related to the promotion of individual and social performance and can play an important role in ensuring the dynamism of any society [3]. According to estimates, 12% of the total global burden of diseases is caused by mental disorders, and also it will keep its increasing trend [4]. According to the reports of the World Health Organization (WHO), the world will witness major changes in the field of epidemiology of diseases and the health needs of humans in the next two decades. Thus, non-communicable diseases (NCDs), such as mental diseases are quickly replaced by infectious and contagious diseases and are at the top of the factors contributing to the disability and premature death [5]. The prevalence of mental disorders in Iran has been reported from 21% to 39.6% [6].

Clinical depression is characterized by behavioral, cognitive, and emotional features. Patients with depression often show signs of dysphoric mood, loss of interest in the normally pleasurable things, self-neglect and social withdrawal, and anorexia or overeating. Insomnia or hypersomnia, fatigue or lack of energy, low self-esteem, difficulty concentrating or making decisions, feelings of hopelessness, sadness, loss of interest or pleasure, feelings of guilt or worthlessness, disturbed sleep or appetite, and lack of concentration [7,8]. Generally, depression affects women more than men and has a global prevalence of 14.4% [9]. In addition, depression can be accompanied by anxiety and suicidal thoughts [10]. Stress is a body's reaction to a stimulus and is usually a short-term experience. Meanwhile, anxiety is a stable mental health disorder that can be caused by stress and negatively affect social, occupational, and other functional domains [11]. Depression is one of the major medical and social issues of the day, and if it continues, the socioeconomic status of sufferers may be witnessed. About 15% of individuals experience a period of major depression disorder at some point in their lives [12].

Anxiety is the anticipation of a future threat. This reaction is different from fear, meaning a reaction to a certain threat. Anxiety is a common feeling in humans and it is compatible with human evolution because by stimulating people to stay away from danger and disasters, it helps them survive [13]. Moreover, anxiety is usually associated with fatigue, lack of energy, confusion, and restlessness. Statistics show that almost 20.8% of Iranian adults suffer from anxiety [14]. Meanwhile, depression, anxiety, and mental distress are the most common mental disorders in the general population. Notably, anxiety and depression are two common mental health diseases. 322 and 264 million individuals suffer from depression and anxiety around the world, respectively [15].

Stress has traditionally been defined as stimuli that befall a person, often called stressors, such as reaction characterized by laboratory shock or loss of job, or physiological arousal and negative effects, especially anxiety [16]. Stress refers to the state of emotional and physiological imbalance between the demands of life and an individual's abilities [17]. Based on the 11th version of the International Classification of Diseases (ICD-11), stress is not considered as a psychological disorder [18], but it may bring about unfortunate consequences, such as decreased self-confidence, feelings of tension, nervousness, indifference, increased irritability, mood swings, sleep disorders, lack of concentration and exhaustion [17].

Stress, anxiety, and depression are related to each other. A meta-analysis study performed in Iran during 1993–2016 reported a prevalence of 40% stress, 42% anxiety, and 44% depression [19]. Age, length of the marriage, level of education, socio-economic status, employment status, place of residence (city and village), marital satisfaction, sleep quality, social support, and physical chronic diseases are among the factors affecting depression, anxiety, and stress [11]. However, it is predicted that one-third to half of the general population may experience mental problems during pandemics such as COVID-19 [20].

However, it is important to identify the source of psychological distress and common mental disorders. Therefore, the objectives of this study were: (1) to investigate t the prevalence of depression, anxiety, and stress among citizens of Karaj, and (2) to predict the prevalence rate by age, gender, marital status, economic status, family size, and education level.

## 2 Materials and methods

### 2.1 Population

This was a population-based, cross-sectional study conducted on all citizens of Karaj.

The study inclusion criteria included individuals over 15 years of age and those living in the Karaj. Participants were excluded from the study if they lived somewhere other than Karaj. Also, restrictions or disabilities that inhibited the individuals to fill out the questionnaire were other exclusion criteria. For instance, individuals were as considered ineligible due to psychosis, cognitive disorders, learning disabilities, or obvious developmental disorder.

According to a study conducted by Montazeri et al. the prevalence of depression in Iran rangeed from 6 to 73% [21]. By considering the results of a study of Mohammadi et al. [22], we assumed that the prevalence of depression was 40% at the 95% confidence level and the accuracy was one-tenth of the prevalence, based on the following formula, the sample size was estimated to be 576.

$$n=\frac{z^{2}\;pq}{d^{2}}=\frac{1.96^{2}*0.4*0.6}{0.04^{2}}=576$$

According to the type of sampling and the design effect 1.5 times more than the sample size, it was estimated that the sample size was 864. Also, 10% was added to this sample to compensate for the non-response rate and missing data. Eventually, 950 individuals were considered as the sample size in this study.

Multi-stage stratified cluster sampling was used to select the participants based on the proportion-to-size approach. Subsequently, the study population was divided into 10 regions based on the standard map of Karaj municipality, and also the population covered by each area was obtained. Then, based on the population of each region, the proportion of the sample size that should be selected from each region was determined. After deciding the sample size in each region, cluster sampling was used. The sample size in each cluster was considered to be 20, so the clustered map was prepared separately for each region, and also the clusters were selected in accordance with the sample size in each center. After identifying each cluster, the sampling team went to the southwest side of this cluster by referring to the address of the cluster and then started sampling the households by moving counter-clockwise and this process continued until the sample size of each cluster was terminated. It should be noted that all family members over 15 years of age were included in the study.

After explaining the objectives of the study and obtaining the written informed consent, two components were studied and collected by the questionnaire. The first part of the questionnaire included demographic information (such as age, gender, race, education, household dimensions, occupation, and insurance coverage) and economic status (including 15 questions about assets was made using principle component analysis as an economic variable).

The second part included the Depression, Anxiety, and Stress Scale (DASS-21) consisting of 21 items. The DASS-21 includes three domains, and also each domain has 7 questions. A 4-point Likert scale ranging from 0 to 3 was used, and also the final score of each domain was obtained through the sum of the scores of the related questions. Toosi et al. previously confirmed the validity and reliability of this tool [23].

### 2.2 Data analysis

To estimate the age and sex-standardized prevalence of the population of Karaj, the total population of the city was determined, and also the proportion of each age-sex group in kataj general population was determined, and then the sample was weighted based on that. Subsequently, the age-sex standardized prevalence of depression, anxiety, and stress was estimated at the 95% confidence interval using binomial distribution. Simple linear regression was first used to check the relationship between the study outcomes and demographic variables. Then multiple linear regression was used for model building and investigating the simultaneous effect of demographic variables on the study outcomes. Notably, a significance level of less than 0.2 was determined for the multiple linear regression of a variable. Besides, the cluster effect was considered in order to correct the standard error. The standardized coefficient was used to determine the most important effective variables in the model. All statistical analysis was performed using Stata version 12 at the 0.05 significance level.

## 3 Results

920 out of 950 participants were willing to continue their cooperation (96.84%). Table 1 shows the distribution of demographic and other variables in the present study. The age mean (SD) of participants was 35.76 (15.69). Also, 458 (49.78%) of participants were female, 437 (47.5) were married, 327 (35.54%) had a bachelor's degree or higher and 304 (33.04%) were classified as a high-level economic group. The 8.03% (6.62–9.45) participants had a history of mental disorder and only 57.02% (54.45–59.59) of participants had the appropriate levels of life expectancy.

**Table 1:** Distribution of demographic variables in study population.

Variables		Frequency (%)	Variables		Frequency (%)
Insurance	cover	786 (85.43)	health service use	Yes	600 (65.22)
	Non-cover	129 (14.02)		No	320 (34.78)
Gender	Male	458 (49.78)	Physician visit	Yes	664 (72.17)
	Female	462 (50.22)		No	254 (27.61)
Marital status	Married	437 (47.5)	Economic status	Low	306 (33.26)
	Single	411 (44.67)		Middle	305 (33.15)
	Divorced -widowed	72 (7.83)		High	304 (33.04)
Education level	<High School Diploma	177 (19.24)	Stress	No	380 (41.35%)
	High School Diploma	306 (33.26)		Moderate	400 (43.53%)
	Associate's degree	110 (11.96)		Severe	139 (15.13%)
	≥Bachelor's degree	327 (35.54)	anxiety	No	487 (52.99%)
family size	1	125 (13.59)		Moderate	310 (33.73%)
	2	122 (13.26)		Severe	122 (13.28%)
	3	205 (22.28)	depression	No	752 (81.83%)
	4	293 (31.85)		Moderate	123 (13.38%)
	≥5	171 (18.59)		Severe	44 (4.79%)
#Age (Yrs. old)		35.76 ± 15.69	#BMI (kg/M^2^)		25.23 ± 4.80

#: presented by mean ± Sd.

As shown in Table 1, the prevalence of severe depression, anxiety, and stress was 4.79%, 13.28% and 15.13%, respectively. The prevalence of moderate depression, anxiety, and stress was 13.38%, 33.73%, and 43.53%; respectively.

Table 2 shows the distribution of mental disorders based on different variables. As shown in Table 2, the prevalence of severe stress, anxiety, and depression in females was 64.03% (56.01–72.05), 54.52% (48.96–60.08), and 59.09% (44.38–73.81), respectively. Based on marital status, the prevalence of severe stress, anxiety, and depression was 10.07% (5.04–15.10), 10.66% (5.15–16.16), and 15.91% (4.96–26.86), respectively. About 36.69% (28.64–44.74) of the participants, who had a bachelor's degree or higher, showed severe stress; however, severe anxiety and severe depression were 27.87% (19.87–35.87) and 40.91% (26.19–55.62), respectively. 69.06% (61.34–76.79) of the participants with severe stress had a history of health service care during the last year, but this value for severe anxiety and severe depression was 68.03% (59.71–76.35) and 59.09% (44.38–73.81), respectively. The prevalence of mental disorders based on other variables is shown in Table 2.

**Table 2:** Distribution of the mental disorders based on different variables.

Variable		Prevalence (95% CI)								
		stress			anxiety			depression		
		No	Moderate	Severe	No	Moderate	Severe	No	Moderate	Severe
Insurance	cover	86.28 (82.81–89.75)	84.85 (81.31–88.39)	87.77 (82.31–93.24)	83.68 (80.38–86.98)	88.64 (85.08–92.19)	87.7 (81.85–93.56)	85.54 (83.02–88.07)	86.18 (80.05–92.31)	90.91 (82.31–99.51)
	\Non-cover	13.72 (10.25–17.19)	15.15 (11.61–18.69)	12.23 (6.76–17.72)	16.32 (13.02–19.62)	11.36 (7.81–14.92)	12.3 (6.44–18.15)	14.46 (11.93–16.98)	13.82 (7.69–19.95)	9.09 (0.49–17.69)
Gender	Male	53.16 (48.13–58.19)	51.25 (46.34–56.16)	35.97 (27.95–43.99)	54 (49.57–58.44)	45.48 (39.92–51.04)	43.44 (34.6–52.29)	50.66 (47.08–54.25)	47.15 (38.28–56.02)	40.91 (26.19–55.62)
	Female	46.84 (41.81–51.87)	48.75 (43.84–53.66)	64.03 (56.01–72.05)	46 (41.56–50.43)	54.52 (48.96–60.08)	56.56 (47.71–65.4)	49.34 (45.75–52.92)	52.85 (43.98–61.72)	59.09 (44.38–73.81)
Marital status	Married	50.01 (44.96–55.04)	46.75 (41.85–51.65)	42.45 (34.19–50.71)	50.51 (46.06–54.96)	44.52 (38.97–50.06)	42.62 (33.8–51.45)	48.27 (44.69–51.85)	46.34 (37.48–55.2)	36.36 (21.97–50.76)
	Single	42.37 (37.39–47.35)	46.01 (41.11–50.91)	47.48 (39.14–55.82)	42.92 (38.51–47.32)	46.77 (41.2–52.34)	46.72 (37.82–55.62)	44.68 (41.12–48.24)	43.9 (35.08–52.72)	47.73 (32.78–62.68)
	Divorced/widowed	7.63 (4.96–10.31)	7.25 (4.73–9.82)	10.07 (5.04–15.10)	6.57 (4.37–8.78)	8.71 (5.56–11.86)	10.66 (5.15–16.16)	7.05 (5.21–8.88)	9.76 (4.48–15.03)	15.91 (4.96–26.86)
Education level	< High School Diploma	21.84 (17.68–26.01)	17.75 (14.02–21.52)	16.55 (10.34–22.75)	18.48 (15.03–21.94)	20 (15.53–24.47)	20.49 (13.29–27.69)	19.28 (16.46–22.11)	18.7 (11.77–25.63)	20.45 (8.38–32.53)
	High School Diploma	30.01 (25.38–34.62)	34.75 (30.07–39.43)	38.13 (30.02–46.24)	33.88 (29.67–38.09)	28.71 (23.66–33.76)	42.62 (33.8–51.45)	33.24 (29.87–36.62)	34.15 (25.72–42.57)	31.82 (17.88–45.76)
	Associate's degree	13.42 (9.98–16.86)	11.75 (8.59–14.91)	8.63 (3.94–13.33)	12.73 (9.76–15.7)	11.94 (8.32–15.56)	9.02 (3.91–14.13)	11.97 (9.64–14.29)	13.82 (7.69–19.95)	6.82 (2.73–14.36)
	≥Bachelor's degree	34.74 (29.94–39.54)	35.75 (31.04–40.46)	36.69 (28.64–44.74)	34.91 (30.66–39.15)	39.35 (33.9–44.81)	27.87 (19.87–35.87)	35.51 (32.08–38.93)	33.33 (24.96–41.71)	40.91 (26.19–55.62)
health service use	Yes	65.53 (60.74–70.32)	63.75 (59.03–68.47)	69.06 (61.34–76.79)	63.86 (59.58–68.14)	66.45 (61.18–71.72)	68.03 (59.71–76.35)	65.16 (61.75–68.57)	68.29 (60.02–76.56)	59.09 (44.38–73.81)
	No	34.47 (29.68–39.26)	36.25 (31.53–40.97)	30.94 (23.21–38.66)	36.14 (31.86–40.42)	33.55 (28.28–38.82)	31.97 (23.65–40.29)	34.84 (31.43–38.25)	31.71 (23.44–39.98)	40.91 (26.19–55.62)
Physician visit	Yes	72.49 (67.97–77.01)	70.75 (66.28–75.22)	76.98 (69.95–84.01)	73.66 (69.74–77.59)	70.87 (65.79–75.95)	71.31 (63.24–79.38)	72.4 (69.19–75.61)	73.98 (66.19–81.78)	68.18 (54.24–82.12)
	No	27.51 (23.01–32.03)	29.25 (24.78–33.72)	23.02 (15.99–30.05)	26.34 (22.41–30.26)	29.13 (24.05–34.21)	28.69 (20.62–36.76)	27.6 (24.39–30.81)	26.02 (18.22–33.81)	31.82 (17.88–45.76)
family size	1	15.83 (12.15–19.52)	12.31 (9.08–15.55)	11.59 (6.23–16.96)	13.99 (10.9–17.08)	14.29 (10.37–18.21)	10.74 (5.2–16.29)	14.42 (11.9–16.94)	12.3 (6.44–18.15)	4.55 (−1.69 to 10.78)
	2	15.83 (12.15–19.52)	12.31 (9.08–15.55)	9.42 (4.52–14.32)	13.58 (10.53–16.63)	12.34 (8.65–16.02)	14.88 (8.5–21.25)	13.48 (11.03–15.94)	10.66 (5.15–16.16)	18.18 (6.64–29.73)
	3	19.26 (15.28–23.24)	23.12 (18.96–27.27)	28.99 (21.38–36.59)	21.19 (17.55–24.84)	25 (20.15–29.85)	20.66 (13.41–27.91)	21.76 (18.8–24.72)	23.77 (16.18–31.37)	29.55 (15.89–43.2)
	4	32.19 (27.47–36.91)	32.66 (28.04–37.28)	29.71 (22.05–37.37)	33.74 (29.53–37.96)	31.17 (25.98–36.36)	27.27 (19.29–35.25)	33.51 (30.12–36.9)	27.05 (19.12–34.97)	20.45 (8.38–32.53)
	≥5	16.89 (13.11–20.67)	19.61 (15.69–23.51)	20.29 (13.55–27.03)	17.49 (14.1–20.88)	17.21 (12.98–21.44)	26.45 (18.54–34.35)	16.82 (14.14–19.51)	26.23 (18.38–34.08)	27.27 (13.94–40.6)
Economic status	Low	32.54 (27.81–37.28)	32.91 (28.29–37.54)	36.96 (28.86–45.05)	29.9 (25.81–33.98)	34.2 (28.88–39.52)	45.08 (36.2–53.96)	31.73 (28.38–35.07)	39.02 (30.36–47.69)	45.45 (30.55–60.36)
	Middle	34.66 (29.85–39.47)	32.16 (27.56–36.76)	33.33 (25.43–41.24)	33.81 (29.59–38.03)	33.55 (28.25–38.85)	31.15 (22.89–39.41)	34.27 (30.86–37.68)	31.71 (23.44–39.98)	22.73 (10.18–35.27)
	High	32.80 (28.06–37.55)	34.92 (30.23–39.62)	29.71 (22.05–37.37)	36.29 (32–40.58)	32.25 (27–37.49)	23.77 (16.18–31.37)	34 (30.6–37.41)	29.27 (21.18–37.35)	31.82 (17.88–45.76)
#Age (Yrs. old)		36.43 (34.76–38.09)	35.24 (33.78–36.7)	35.38 (32.78–37.98)	35.92 (34.5–37.35)	36.15 (34.42–37.87)	34.08 (31.42–36.74)	35.79 (34.66–36.92)	35.84 (33.05–38.63)	34.82 (30.61–39.03)
#BMI (kg/M^2^)		25.22 (24.76–25.68)	25.19 (24.7–25.68)	25.34 (24.54–26.14)	25.3 (24.88–25.73)	25.02 (24.5–25.54)	25.44 (24.49–26.4)	25.24 (24.9–25.58)	25.31 (24.33–26.29)	24.75 (23.7–25.81)

CI: Confidence Interval.

#: presented by mean (95% CI).

Table 3 shows the association of stress, anxiety, and depression with demographic variables by simple linear regression. Hence, there stress was positivelyassociated with being female (b: 2.09; p: <0.001), and being divorced or widowed divorce/being a widow (b: 1.74; p: 0.006). However, a negative association was observed between stress and the education level higher than a bachelor's degree (b: 1.21; p: <0.001) and high economic status (b: 1.81; p: <0.001). A similar pattern was shown for anxiety as anxiety was positively associated with being female (b: 1.36; p: <0.001), and being divorced or widowed (b: 1.56; p: 0.031), but a negative association was observed between the education level higher than a bachelor's degree (b: 2.02; p: <0.001), high economic status (b: 1.68; p: <0.001) and family size (b: 1.17; p: <0.001). As shown in Table 3, being divorced or widowed (b: 1.87; p: 0.048), a bachelor's degree (b: 0.58; p: 0.031), high economic status (b: 1.83; p < 0.001), and age (b: 1.50; p: 0.002) were significantly associated with depression.

**Table 3:** Association of stress, anxiety and depression with demographic variables by simple linear regression.

Variables		Stress score		Anxiety score		Depression score		Total score	
		Coefficient (95% CI)	p-value	Coefficient (95% CI)	p-value	Coefficient (95% CI)	p-value	Coefficient (95% CI)	p-value
Insurance	cover	**Reference**	**—**	**Reference**	**—**	**Reference**	**—**	**Reference**	**—**
	no-cover	−0.58 (−2.14 to 0.97)	0.461	−0.54 (−1.82 to 0.75)	0.411	−0.65 (−2.06 to 0.77)	0.373	−1.77 (−5.52 to 1.99)	0.356
Gender#	Male	**Reference**	**—**	**Reference**	**—**	**Reference**	**—**	**Reference**	**—**
	Female	2.09 (1.02–3.16)	<0.001*	1.36 (0.48–2.25)	<0.001*	0.57 (−0.41 to 1.55)	0.262	4.02 (1.43–6.61)	0.002*
Marital status#	Married	**Reference**	**—**	**Reference**	**—**	**Reference**	**—**	**Reference**	**—**
	Single	0.84 (−0.28 to 1.96)	0.142	0.58 (−0.35 to 1.51)	0.223	0.56 (−0.46 to 1.58)	0.286	1.99 (−0.72 to 4.69)	0.151
	Divorced/Widowed	1.74 (0.34–3.81)	0.006*	1.56 (0.16–3.01)	0.031*	1.87 (0.01–3.75)	0.048*	5.16 (0.15–10.16)	0.044*
Education level #	< High School Diploma	**Reference**	**—**	**Reference**	**—**	**Reference**	**—**	**Reference**	**—**
	High School Diploma	−0.3 (−2.28 to 1.68)	0.765	0.22 (−1.06 to 1.5)	0.733	0.36 (−1.04 to 1.76)	0.623	1.79 (−1.93 to 5.52)	0.345
	Associate's degree	−0.8 (−2.32 to 0.73)	0.306	−1.31 (−1.95 to −0.84)	0.001*	−0.29 (−2.1 to 1.51)	0.754	−0.9 (−5.69 to 3.89)	0.712
	≥Bachelor's degree	−1.21 (−2.98 to −0.33)	<0.001*	−2.02 (−3.28 to −1.25)	<0.001*	−0.58 (−0.99 to −0.10)	0.031*	−1.36 (−2.32 to −0.15)	0.001*
Health service use	Yes	**Reference**	**—**	**Reference**	**—**	**Reference**	**—**	**Reference**	**—**
	No	−0.22 (−1.35 to 0.92)	0.709	−0.32 (−1.26 to 0.62)	0.499	0.36 (−0.67 to 1.38)	0.503	−0.18 (−2.92 to 2.55)	0.895
Physician visit	Yes	**Reference**	**—**	**Reference**	**—**	**Reference**	**—**	**Reference**	**—**
	No	−0.16 (−1.37 to 1.05)	0.792	0.16 (−0.84 to 1.16)	0.753	0.1 (−0.99 to 1.2)	0.850	0.1 (−2.81 to 3.02)	0.944
Economic status#	Low	**Reference**	**—**	**Reference**	**—**	**Reference**	**—**	**Reference**	**—**
	Middle	−1.83 (−2.15 to −0.50)	<0.001*	−1.08 (−2.17 to 0.02)	0.051	−1.72 (−2.92 to −0.53)	0.011*	−3.63 (−6.81 to −0.44)	0.026*
	High	−1.81 (−2.14 to −0.47)	<0.001*	−1.68 (−2.78 to −0.59)	<0.001*	−1.83 (−3.03 to −0.63)	<0.001*	−4.32 (−7.51 to −1.13)	0.008*
BMI (kg/M^2^)		−0.02 (−0.13 to 0.09)	0.710	0.01 (−0.08 to 0.1)	0.813	−0.01 (−0.11 to 0.1)	0.904	−0.02 (−0.29 to 0.26)	0.908
Family size#		0.35 (−0.06 to 0.77)	0.097	−1.17 (−1.84 to −0.87)	<0.001*	0.37 (−0.01 to 0.75)	0.063	0.89 (−0.12 to 1.91)	0.083
Age (Yrs. old)		−0.02 (−0.06 to 0.01)	0.222	−0.02 (−0.05 to 0.01)	0.252	1.50 (0.03–2.87)	0.002*	2.04 (1.05–3.14)	0.001*

*: significant at 0.05.

#: eligible for including to multiple linear regression.

As mentioned earlier, for model building, only variables that had a significant association with mental disorders in the simple analysis were eligible for including in the multiple models. Table 4 shows the association between mental disorders and the study variables using multiple ordinal linear regression models. However, this model is more valuable for the interpretation of the results.

**Table 4:** Association between the mental disorders with different variables of multiple linear regression.

Variables		Stress score		Anxiety score		Depression score		Total score	
		Coefficient (95% CI)	p-value	Coefficient (95% CI)	p-value	Coefficient (95% CI)	p-value	Coefficient (95% CI)	p-value
Gender#	Male	Reference	**—**	Reference	**—**	NI		Reference	**—**
	Female	2.05 (0.97–3.12)	<0.001*	1.01 (0.28–1.91)	0.002*			3.99 (1.31–6.25)	0.001*
Marital status#	Married	Baseline	**—**	Reference	**—**	Reference	**—**	Reference	**—**
	Single	0.77 (−0.35 to 1.89)	0.142	0.62 (−0.40 to 1.71)	0.321	0.58 (−0.49 to 1.62)	0.301	2.01 (−0.69 to 4.54)	0.184
	Divorced/Widowed	1.84 (1.29–2.65)	0.005*	1.21 (0.33–1.64)	0.001*	1.85 (0.25–3.49)	0.003*	5.17 (0.20–10.99)	0.041*
Education#	< High School Diploma	Reference	**—**	Reference	**—**	Reference	**—**	Reference	**—**
	High School Diploma	−0.3 (−2.28 to 1.68)	0.765	0.14 (−1.24 to 1.74)	0.871	0.36 (−1.11 to 1.85)	0.523	1.81 (−1.95 to 5.57)	0.366
	Associate's degree	−0.8 (−2.32 to 0.73)	0.306	−1.11 (−1.84 to −0.64)	0.001*	−0.32 (−2.12 to 1.61)	0.854	−0.92 (−5.72 to 3.94)	0.641
	≥Bachelor's degree	−1.21 (−2.98 to −0.33)	<0.001*	−2.03 (−3.30 to −1.23)	<0.001*	−0.48 (−1.18 to −0.02)	0.039*	−1.16 (−2.21 to −0.23)	0.001*
Economic status#	Low	Reference	**—**	Reference	**—**	Reference	**—**	Reference	**—**
	Middle	−1.83 (−2.15 to −0.50)	<0.001*	−1.09 (−2.13 to −0.01)	0.049*	−1.71 (−2.91 to −0.56)	0.009*	−3.61 (−6.80 to −0.45)	0.021*
	High	−1.81 (−2.14 to −0.47)	<0.001*	−1.70 (−2.72 to −0.45)	<0.001*	−1.80 (−3.01 to −0.60)	<0.001*	−4.31 (−7.52 to −1.15)	0.009*
family size #		0.39 (0.04–0.82)	0.041*	−1.16 (−1.80 to −0.83)	<0.001*	NI		NI	
Age (Yrs. old)		NI		−0.02 (−0.04 to 0.02)	0.264	1.53 (0.06–2.70)	0.003*	2.15 (1.16–3.11)	0.001*

*: significant at 0.05.

#: eligible for including to multiple linear regression.

NI: not included.

As shown in Table 4, there was a positive association among being female, stress (b: 2.05; p < 0.001), anxiety (b: 1.01; p: 0.002), and the total score (b: 3.99; p: 0.001) adjusted for other variables. In other words, the mean score of different mental health domains in females was more significant than that in males. Also, there was a positive association between being divorced or widowed and mental disorders as the score of stress (b: 1.84; p: 0.005), anxiety (b: 1.21; p: 0.001), depression (b: 1.85; p: 0.003), and the total score (b: 5.17; p: 0.041) in divorced/widow participants were higher than those in married ones. The association of education level with stress (b: 1.21; p < 0.001), anxiety (b: 2.03; p < 0.001), depression (b: 0.48; p: 0.039), and the total score (b: 1.16; p: 0.001) remained significant. However, the score of the mentioned domain in the participants who had a bachelor's degree or higher was lower than those who earned the education under a high school diploma or an associate's degree. Also, the results of multiple linear regression analysis showed that higher economic status was negatively associated with mental disorders. However, the mean score of stress, anxiety, depression, and the total score, which was 1.81 (p < 0.001), 1.70 (p < 0.001), 1.80 (p < 0.001), and 4.31 (p < 0.001), respectively, in higher economic status was lower than that in lower economic status. This pattern was also true for middle economic status as the score of mental disorder domains in the middle economic status group was lower than that in lower economic status group.

Our analysis also showed that the increase in family size was positively associated with stress (b: 0.39; p: 0.041), but there was a negative association between family size and anxiety (b: 1.16; p < 0.001). In other words, large households showed a higher stress level but a lower anxiety level. There was no association between family size and depression as well as the total score.

Also, a standardized coefficient was estimated for determining the most effective variables in each model. As shown in Table 4, the most effective variable for stress was gender (standardized beta: 0.135). With regard to anxiety, depression, and total score, economic status was a more effective variable in multiple models (standardized beta: 0.127, −0.117, and −0.112; respectively) (Table 5).

**Table 5:** Standardized coefficients for determining the most effective variables in outcomes.

Variables	Score			
	Stress	Anxiety	Depression	Total
Gender	0.135	0.104	–	0.107
Marital status	0.066	0.049	0.060	0.059
Education level	0.061	0.047	0.068	0.061
Economic status	−0.066	−0.127	−0.117	−0.112
Family size	0.066	0.041	–	–
Age		−0.037	−0.007	−0.031
NI: Not Included				

## 4 Discussion

The results of the present study demonstrated that education level was associated with depression, stress, and anxiety. Seemingly, it was found that low information about mental disorders like depression can lead to worsening the symptoms [24]. However, it was proved that stress, depression, and anxiety are more prevalent among women compared with men. This finding is in line with results of previous studies conducted in Iran, showing females are more vulnerable to psychiatric disorders [25]. Also, such disorders are more prevalent in women compared with men in other countries [26]. Socioeconomic disadvantages, sex hormones, cultural differences, and violence are among the effective variables in the higher prevalence of psychiatric disorders in females. Besides, women are more inclined toward expressing their emotions and feelings [27]. Also, women are more prone to experience PTSD [28]. However, one study claimed that there was no significant difference between males and females in terms of depression [29].

Another finding obtained from this study showed that being divorced or widowed was directly related to the incidence of anxiety, depression, and stress, which was in line with the findings of a study, suggesting a higher incidence of depression in single or divorced individuals as they experience more loneliness [30]. However, a study claimed that the prevalence of anxiety, depression, and stress was lower in the non-married population. It is argued that the quality and duration of marriage play an important part in this regard [31].

Also, many researches focused on comorbidity between these three disorders [32]. Similarly, a study proved that the simultaneous incidence of post-traumatic stress disorder (PTSD) and depression was more common compared with sole occurrence of PTSD or depression [33]. The comorbidity of anxiety, depression, and stress was not evaluated in the studies, but a study evaluated the comorbidity of PTSD, anxiety and depression [34].

Particularly, the prevalence of psychiatric disorders is reported with fluctuating data in Iran. For instance, national surveys in 2001, 2002, 2012, and 2021 reported that the prevalence of psychiatric disorders was 21.5%, 34.3%, 39.7%, and 31.03%, respectively [35]. The fluctuating results are due to the presence of various assessment tools and inventories. It therefore is recommended to design preventive programs in the childhood of prone individuals in order to reduce the prevalence of psychiatric disorders among the adult population [36]. Anxiety disorders are the most common disorders in children and adolescents.10–20% of this population suffer from one or even more kinds of anxiety disorders [37]. The anxiety can lead to academic and social pressures. Adolescents with high anxiety level can suffer from disorders, such as lack of concentration in-class work, excessive worry and nervousness, headache, gastrodynia, and reduced attention [38]. Meanwhile, the unemployment of parents can be an effective factor in affecting the mental health of teenagers in a negative way [39]. It is proved that the unemployment of parents can lead to at least one-day of hospitalization in their children [39]. Seemingly, depression is a common disorder among both children and adults which female gender, family history, impaired cognitive cognition and unpleasant events are among the risk factors [40]. It is reported that individuals aged 15 to 18 are more prone to depression due to the factors, such as the competitive atmosphere of classes and the stress related to it [41].

Other findings of this study showed that women are more vulnerable to depression, anxiety, and stress. Similarly, special attention should be paid to pregnant women as the prevalence rate of anxiety and depression has increased among them after COVID-19 pandemic [42]. Other studies also showed that women are more vulnerable to stress [43]. As mentioned above, a lower educational level in pregnant women was associated with a higher prevalence of anxiety and depression [44]. However, another study showed that a higher level of education was associated with a higher prevalence of depression, anxiety, and stress [45]. However, low maternal education level is considered as one of the risk factors for psychiatric disorders in children [46]. Other factors, such as unemployment, playing the role of a housewife, a sedentary lifestyle, and face-to-face follow-up visits can contribute to increasing the prevalence rate of anxiety and depression [47].

Isolations originating from the COVID-19 pandemic also led to a higher incidenceof anxiety, stress, and depression. A study on the Chinese population showed that the prevalence of anxiety, stress, and depression was 28.8%, 53.8%, and 16.1%, respectively [48]. One of the main factors, leading to a higher prevalence of depression and suicidal thoughts, is related to unemployment. Similarly, the level of suicide is hugely increased during mass unemployment [49]. Another study has also shown that retired individuals are more prone to stress, anxiety, and depression [43]. The association between stress and unemployment cannot be fully confirmed as one study proved that only 10.4% of the participants suffered from a severe levels of stress during their unemployment [50]. Meanwhile, the highlighted role of economic stressors in forming anxiety should not be neglected [51].

### 4.1 Limitations

Although the research team made a lot of effort to complete the study, the present study had some limitations. Due to the cross-sectional nature of the study, the observed relationships cannot be considered causal. Also, due to the cross-sectional nature of the study, we could not examine other influential factors. On the other hand, one of our limitations was the lack of funds because we wanted to examine the status of other mental disorders, which was not possible due to budget limitations. The high sample size, the use of a trained questioning team, and the random selection of a representative sample from the population of Ilam were among the strengths of this study.

## 5 Conclusion

The current study could expand the knowledge on the prevalence of anxiety, depression, and stress in Karaj population. The multiple linear regression showed that age, being female, being divorced or widowed, and having a bachelor's degree or lower were associated with the incidence of depression, anxiety, and stress. However, the family size was more associated with stress. It was proved that the higher the economic status, the lower the presence of depression, stress, and anxiety.

Further research is needed in these areas. It is hoped this study would provide useful information on the prevalence of depression, anxiety, and stress. Given that some participants in this study experience levels of depression, anxiety, and stress, which may have negative effects on their family life, it is therefore important to pay attention to this problem and it should be a clinical priority. It is also recommended to conduct studies that reduce future problems and possible challenges that may originate from the studies of mental disorders.

## Ethical approval

The study protocol was approved by the Ethics Committee of Alborz University of Medical Sciences (No. IR.ABZUMS.REC.1400.295**).** Also, the written informed consent was obtained from all the participants.

## Funding information

This study was not supported by any funding.

## Author contribution statement

Sara Armandpishe; Fatemeh Abdi: Conceived and designed the experiments; Wrote the paper. Maryam Sardashti; Kimia Soltaniha: Performed the experiments. Reza Pakzad: Analyzed and interpreted the data. Mohammadamin Jandaghian-Bidgoli: Contributed reagents, materials, analysis tools or data; Wrote the paper.

## Data availability statement

Data included in article/referenced in article.

## Declaration of competing interest

The authors state no conflicts of interest in this study.
